# A bibliometric analysis and visualization of non-suicidal self-injury in adolescents

**DOI:** 10.3389/fpsyt.2024.1457191

**Published:** 2024-11-11

**Authors:** Jingtong Luo, Xueru Yang, Hongli Li, Lin Fan, Xuehe Chen, Jiayi Li, Tianming Song

**Affiliations:** School of Nursing, China Medical University, Shenyang, China

**Keywords:** adolescent, non-suicidal self-injury, bibliometrics, visualization analysis, NSSI

## Abstract

**Background:**

Non-suicidal self-injury(NSSI)is a widespread occurrence among adolescents, and this behavior can bring serious consequences. In recent years, the prevalence of NSSI continues to rise, which has attracted the attention of many researchers. But currently there is no research exploring the overall research distribution of NSSI in adolescents through quantitative analysis. Therefore, it is necessary to understand the status of development and main research hotspots of NSSI in adolescents via bibliometric analysis.

**Methods:**

We searched the relevant studies from the Web of Science Core Collection(WoSCC)from January 1, 2014 to December 31, 2023. Using CiteSpace and VOSviewer visual analysis tools, we analyzed studies from the perspectives of country, region, institution, journal, author, and keywords.

**Result:**

A total of 2177 studies related to NSSI in adolescents were included. USA and Harvard University were the leading country and institution in this research field. Penelope Hasking was the most prolific author. *Frontiers in Psychiatry* and the *Journal of Affective Disorders* were the most productive journals. The most high-frequency keywords were ‘depression’, ‘mental health’, ‘emotion regulation’ and ‘borderline personality disorder’. ‘mindfully’, ‘intervention’, ‘self-compassion’ and ‘ecological momentary assessment’ were the emerging keywords.

**Conclusions:**

Exploring the relevant factors and mechanisms of comorbidities, identifying etiology and risk/protective factors, and finding the impact of NSSI on adolescents are the hot topics. Moreover, intervention measures and interdisciplinary collaborative research for NSSI in adolescents will emerge as frontiers in the future.

## Introduction

Non-suicidal self-injury (NSSI) is defined as the act of directly and intentionally injuring oneself without suicidal intent, including cutting, burning, hitting, scalding, biting oneself, and other forms (1), and behaviors that cause injury but are socially acceptable, such as piercing or body modification, are excluded (2). NSSI most commonly occurs in early to mid-adolescence, adolescence is a vulnerable phase for developing NSSI (3). Although adolescents mature physically during adolescence, they have not yet reached psychological maturity. As such, adolescents are more impulsive, may have difficulty regulating negative emotions, and will be prone to NSSI behaviors (4). The prevalence rate of NSSI in adolescents can reach 22%, which is seven times higher than for all age groups (5). A meta-analysis also indicated that the lifetime prevalence of NSSI in adolescents was 22.1%, and the 12-month prevalence was 19.5% (6). In clinical samples, the lifetime prevalence could be as high as 30% to 82% (7). Moreover, the COVID-19 pandemic in late 2019 has increased the incidence rate of NSSI and the number of NSSI behaviors in adolescents (8).

NSSI might lead to suicide attempts or suicide behavior (9); 18% of children and adolescents had suicidal ideation in their lifetime, and 6% had suicide attempts in the past 12 months (6). Notably, suicide is the fourth leading cause of death among 15–29-year-olds (9). Thus, the widespread occurrence and deleterious effects of NSSI have garnered global attention. A longitudinal cohort study pointed out that compared to adolescents who did not participate in NSSI, adolescents who had NSSI behaviors would have a higher longitudinal risk of alcohol/substance use disorders and psychiatric hospitalization (10). Similarly, a survey conducted among college students showed that even after controlling for pre-existing psychological disorders, NSSI still predicted future psychological disorders and predicted that adolescents would suffer from generalized anxiety disorder and bipolar disorder (11). Recently, the body of research on NSSI has rapidly grown (3), and the fields involved have become increasingly broad, no longer confined to psychiatry but also psychology (12) and sociology (13). Therefore, gaining a deeper comprehension of the global hot trends in adolescent NSSI research holds immense importance for those actively involved in NSSI-related research and can better promote the knowledge exchange and development of this field in the future.

Bibliometric analysis is a scientific and quantitative research method of publications, including co-word analysis, social network analysis, and cluster analysis to summarize the progress of a research theme. Quantitative statistics can detect the hotspots or emerging trends and contributions of authors, journals, institutions, or countries (14). Notably, bibliometrics is a powerful instrument that leverages data mining, information processing, statistical analysis, and mapping techniques to delve into existing research (15). By utilizing bibliometrics, scholars can gain a visual understanding of the internal knowledge structure and evolution process within a discipline, enabling scholars to understand the academic achievements in their research field and determine future research direction (16). CiteSpace and VOSviewer are two Java-based information visualization software that are widely used in the field of bibliometrics (17). Furthermore, CiteSpace and VOSviewer can analyze countries/regions, institutions, authors, and keywords, thus providing a comprehensive interpretation of the development process of the research field (18).

At present, there has been no bibliometric analysis of NSSI in adolescents. Therefore, we used bibliometrics and literature visualization tools to analyze the global research trend of NSSI in adolescents. The visual map presents the outcomes, enabling a deeper exploration of the research hotspots, anticipated trends, and potential applications of NSSI in adolescents.

## Methods

### Design

In this study, bibliometric analysis was performed on studies published within the scope of NSSI in adolescents. The bibliometric analysis was used to evaluate the trends of the studies, mapping the latest developments, collaborations, and citation analyses by visualizing them with social network analysis.

### Data sources

The Web of Science Core Collection (WOSCC) database, which is compatible with the CiteSpace program, was used to obtain the research data. WOSCC, one of the most accepted databases, is an important database for analyzing scientific publications, providing access to citation statistics and bibliographic data of publications. Thus, data were extracted from the WOSCC database with the “Save in other file formats and full records and cited references” option. Moreover, Microsoft Office Excel and Endnote programs were used to manage the data.

### Search strategy

The literature was searched online using the WoSCC. All data were obtained on 2 February 2024 to avoid bias caused by database updates, yielding 5973 results. The time span was from 1 January 2014 to 31 December 2023. The following search terms were used: TS=(((“self-harm” OR “self injure*” OR “self mutilat*” OR “self-abuse” OR “self-attack”) NOT “suicide*”) OR (“non-suicidal self-injury” OR “NSSI”) AND (“adolescen*” OR “teen*” OR “student*” OR “youth” OR “young” NOT “adult”)).

### Inclusion/exclusion criteria

**Figure 1:**
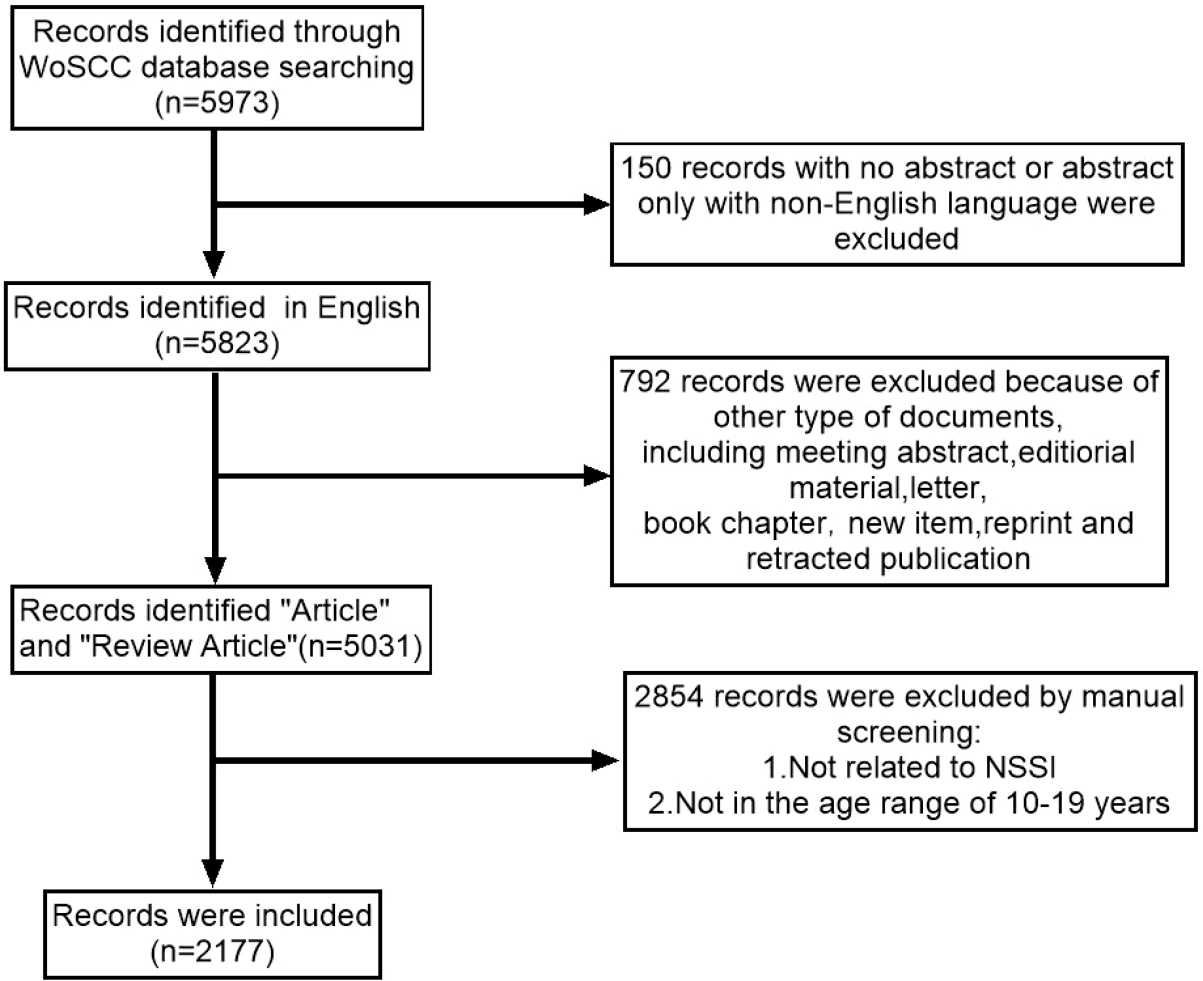
Flow chart of literature screening.

Studies were included if they (1) were related to adolescents (10–19 years old) (19) with NSSI (2), were articles and reviews, and (3) were in English. Two researchers independently reviewed titles and abstracts and removed studies unrelated to NSSI in adolescents. A total of 2177 publications were retained after reviewing titles and abstracts, as shown in **Figure 1**.

### Analysis tool

CiteSpace is a Java-based visualization software developed by Professor Chaomei Chen in 2004 for analyzing and visualizing citation networks in scientific literature (20). In this study, the records retrieved from the WoSCC database were downloaded and exported in plain text format, including full records and citations, with the file named “download_XXX.” Subsequently, the file was imported into CiteSpace for bibliometric and visual analysis. The specific parameters used in CiteSpace were as follows: Parameter Settings: Time slice: January 1, 2014 - December 31, 2023; Year per slice: 1 year; Selection criteria: Top N = 50. All other settings were left as the software defaults. Different nodes on the generated map represent various entities, such as countries, institutions, or journals. A node’s size represents the publication’s centrality or frequency, with larger nodes indicating higher occurrences or citation frequency (18). The links between nodes indicate collaborative relationships, co-authorship, or co-citation networks. The colors of nodes and links represent different clusters (18).

VOSviewer is a free software tool introduced in 2009 by the research group of Nees J. Van Eck at Leiden University in the Netherlands (21). It was designed to build and visualize scientific knowledge networks based on similar relationships. VOSviewer provides visual analysis and creates maps based on network data, enabling the construction of network maps for academic publications, scientific journals, authors, research institutions, countries, and keywords (17). The visualization map generated by VOSviewer displays keywords or terms in the data file according to a specific clustering technique. This technique is derived from the principles of mapping and clustering, and is based on the VOS mapping technique. At the same time, the clustering technology of VOSviewer also employs a special clustering method, which can be seen as a weighted variant of modular-based clustering, with resolution parameters for identifying small clusters. Correspondingly, the visualization map of term occurrence depicts the frequency of occurrence of certain key terms, and terms are represented as nodes of different sizes and colors, the size of which is proportional to the frequency of term occurrence, the nodes’ color reflect the terms’ clustering (22). These terms are connected into a visual network through citation, co-occurrence, citation, and bibliographic coupling. The color and thickness of the lines between nodes in the network represent different clusters and correlation strengths. This study imported the retrieved data into VOSviewer in plain text format for analysis by country, institution, author, and keywords. The VOSviewer software parameters were set as follows: Minimum publication thresholds for countries and authors: 5 and 10, respectively; Minimum citation threshold for journals: 15; Minimum keyword occurrence threshold: 10.

## Results

### Annual global publication outputs

**Figure 2:**
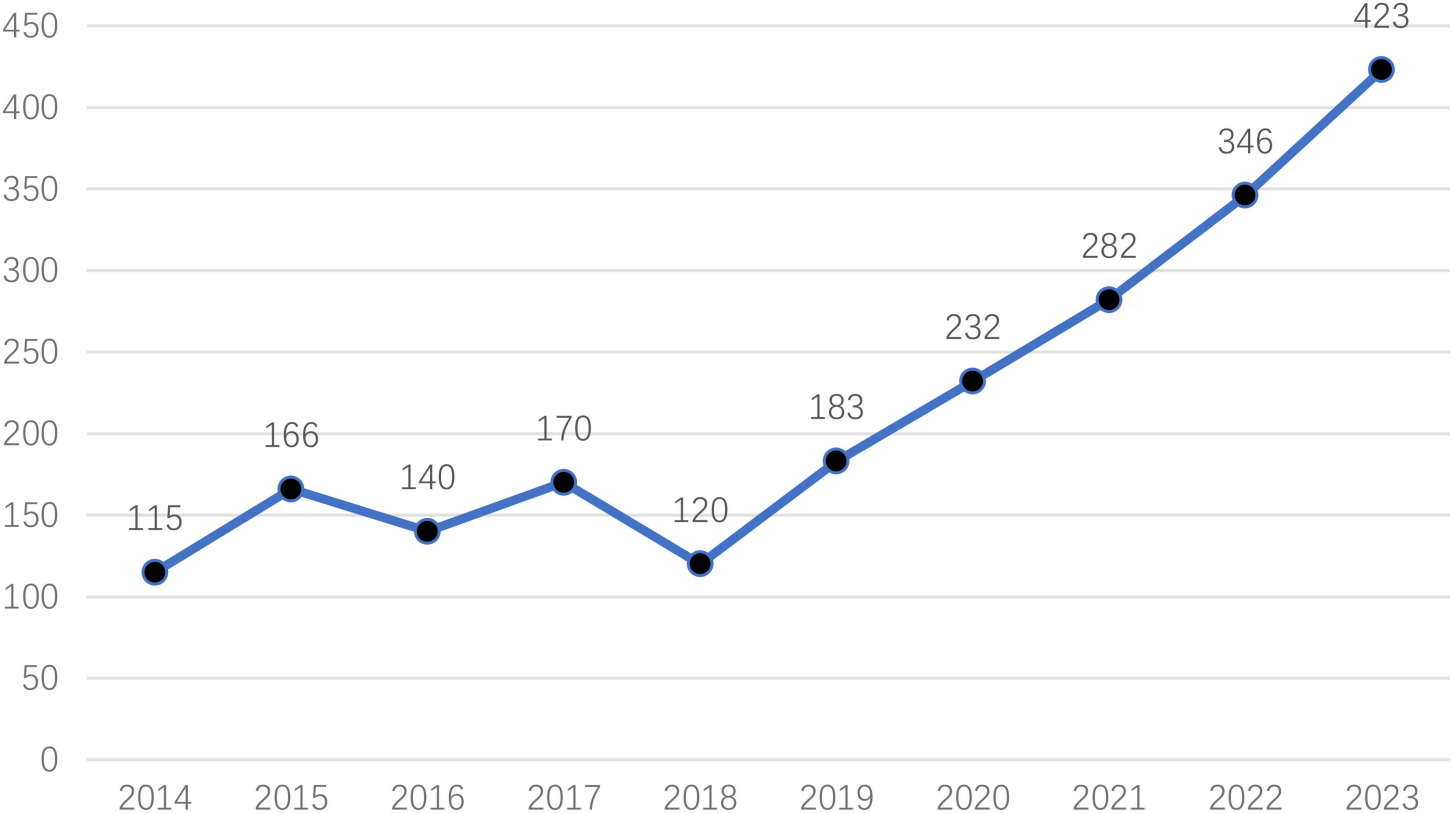
Number of publications on NSSI in adolescents by year.

The output of annual publications is shown in **Figure 2**. The number of articles in the field of NSSI in adolescents fluctuated between 100 and 200 from 2014 to 2018. The progress of research in the field of NSSI in adolescents is relatively stable. Since 2018, the number of publications has skyrocketed. Moreover, the annual global publications increased from 120 in 2018 to 423 in 2023, an increase of 352.5%. Therefore, this indicates that more researchers are becoming interested and involved in this field, and the number of publications in the future will continue to increase.

### Publication analysis based on countries

NSSI is a subject of interest throughout the world, with a total of 69 countries/regions contributing to NSSI in adolescents. **Table 1** lists the top 10 most productive countries regarding the number of publications. In terms of national research strength, the USA was the most productive country(n=811), and the number of publications far exceeds that of other countries, followed by China(n=345) and England(n=261). Although China had many publications, its centrality was relatively low compared to the USA and England, and China lacked international cooperation with other countries.

**Table 1:** The top 10 productive countries/regions.

Ranking	Country	Publications	Percent	Centrality
1	USA	811	28.25%	0.53
2	China	345	12.02%	0.02
3	England	261	9.09%	0.31
4	Canada	207	7.21%	0.04
5	Australia	181	6.30%	0.05
6	Germany	132	4.60%	0.04
7	Sweden	90	3.13%	0.01
8	Belgium	83	2.89%	0.01
9	Italy	73	2.54%	0.01
10	Spain	56	1.95%	0.01

**Figure 3:**
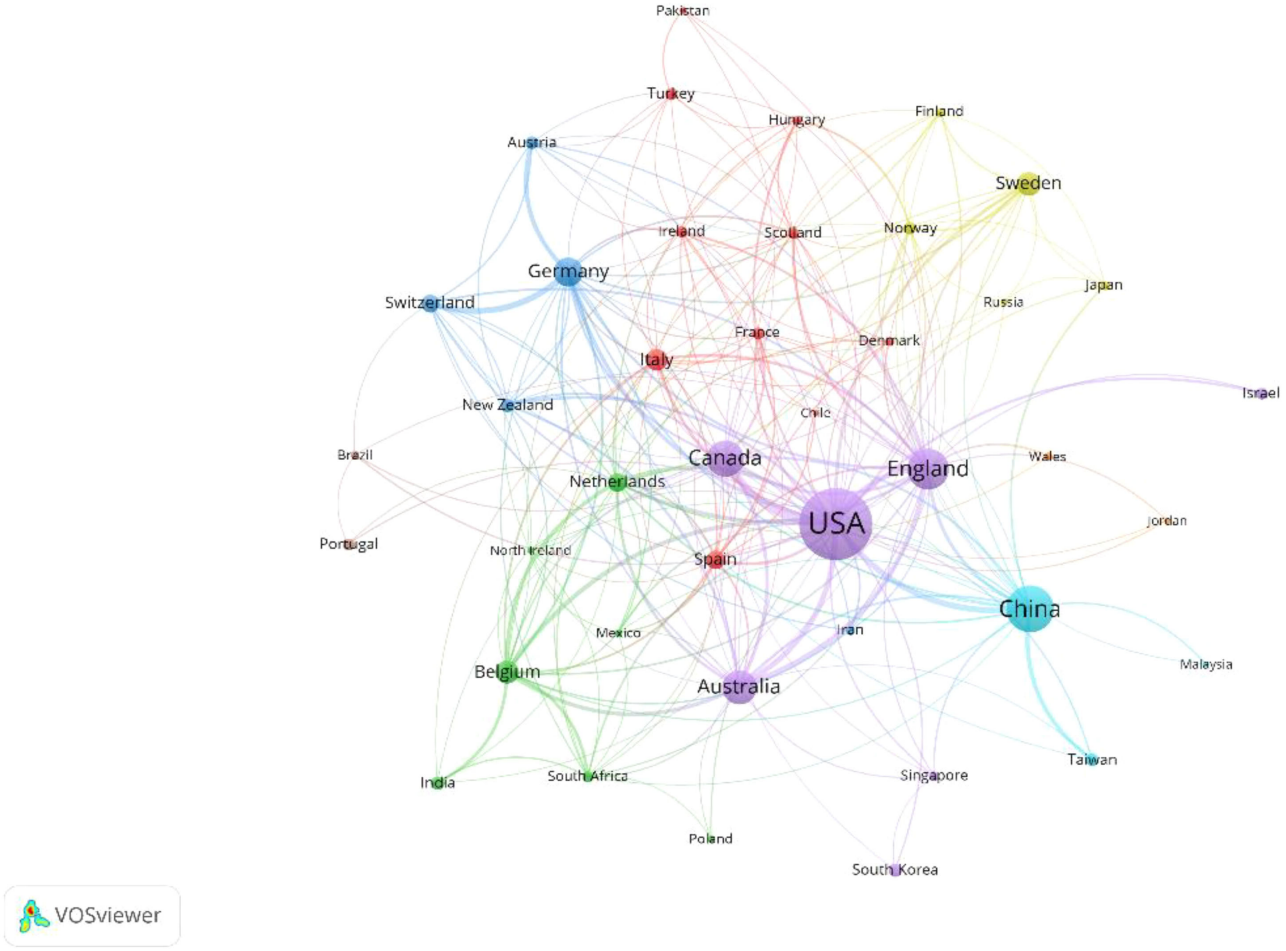
The co-authorship network of countries/regions.

VOSviewer was used for co-authorship analysis of the countries/regions to reveal international collaborations in this field. The co-authorship network of countries/regions is shown in **Figure 3**. The co-authorship network, including 69 countries/regions, was divided into six clusters represented by different colors. The largest cluster (purple), consisting of 7 countries, centers around the USA, England, Canada, and Australia. Notably, the USA had the most significant number of cooperating partners (n = 37), followed by England (n = 31), Germany (n = 24) and Canada (n = 24).

### Publication analysis based on institutions

The publications included in the bibliometric analysis were published with contributions from 2171 institutions. The top 10 productive institutions are listed in **Table 2**. Harvard University (n = 105) had the most publications, followed by Curtin University (n = 83) and University of London (n = 80).

**Table 2:** The top 10 productive institutions.

Ranking	institutions	publications	centrality
1	Harvard University	105	0.16
2	Curtin University	83	0.05
3	University of London	80	0.07
4	Pennsylvania Commonwealth System of Higher Education (PCSHE)	75	0.05
5	KU Leuven	66	0.09
6	South China Normal University	60	0.04
7	Ruprecht Karls University Heidelberg	59	0.1
8	Brown University	52	0.2
9	University System of Ohio	49	0.06
10	University of California System	47	0.13

**Figure 4:**
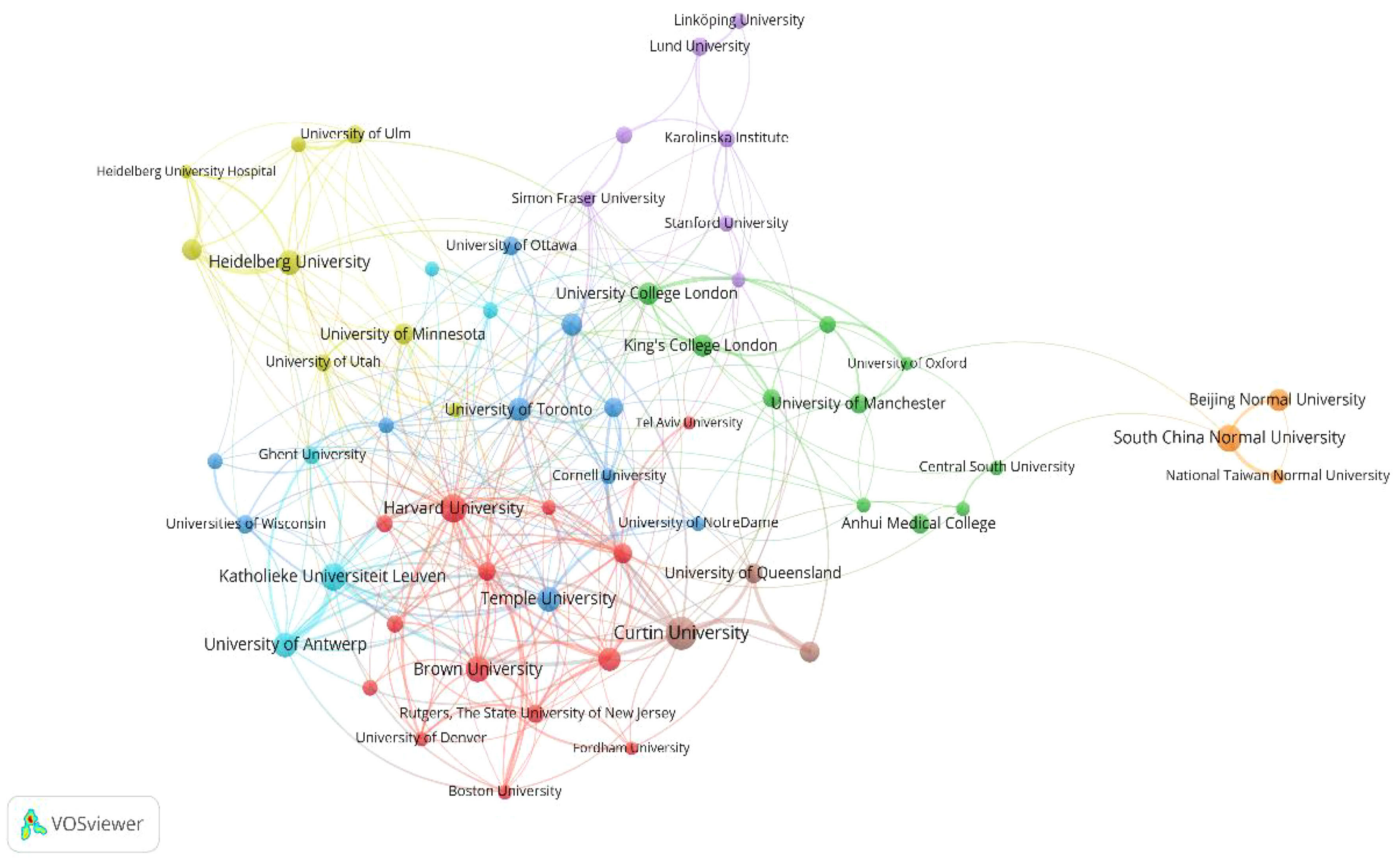
The co-authorship network of institutions.

When the minimum number of articles published by the institutions was set to 15, a total of 60 institutions met the criteria. VOSviewer was utilized for co-authorship analysis of these 60 productive institutions. The co-authorship network of institutions is revealed in **Figure 4**. The co-authorship network, including 60 institutions, was divided into 8 clusters represented by different colors. The red cluster of 14 institutions centered on Harvard University and Brown University was the largest. Moreover, Harvard University had the most significant number of cooperating partners (n = 33), followed by Curtin University (n = 20) and Brown University (n = 19).

### Publication analysis based on journals

The retrieved articles on NSSI in adolescents were published in 852 journals. **Table 3** lists the top 10 journals that published the most articles on NSSI in adolescents and accounted for 27.7% (604/2177) of the publications. Frontiers in Psychiatry and Journal of Affective Disorders were the most productive journals (92 publications), followed by Psychiatry Research with 85 publications and Archives of Suicide Research with 77 publications.

**Table 3:** The top 10 productive journals.

Ranking	Journal	Publications	IF (2022)	JCR
1	*Frontiers in Psychiatry*	92	4.7	Q2
2	*Journal of Affective Disorders*	92	6.6	Q1
3	*Psychiatry Research*	85	11.3	Q1
4	*Archives of Suicide Research*	77	2.8	Q3
5	*Suicide and Life-threatening Behavior*	65	3.2	Q2
6	*Journal of Clinical Psychology*	47	3	Q2
7	*BMC Psychiatry*	46	4.4	Q2
8	*Child and Adolescent Psychiatry and* *Mental Health*	37	5.6	Q1
9	*International Journal of Environmental* *Research and Public Health*	36	4.614	Q2
10	*Current Psychology*	27	2.8	Q2

**Table 4** lists the top 10 highly cited journals. *Psychiatry Research* ranked third in terms of publications (n = 85), was the journal with the most citations (n = 3290), followed by *Suicide and Life-threatening Behavior*, which ranked fifth in terms of publications (n = 65), with 2772 citations and *Journal of Affective Disorders*, ranked first in terms of publications (n = 92), with 2429 citations.

**Table 4:** The top 10 highly cited journals.

Ranking	Cited Journal	Citations	IF (2022)	JCR
1	*Psychiatry Research*	3290	11.3	Q1
2	*Suicide and Life-threatening Behavior*	2772	3.2	Q2
3	*Journal of Affective Disorders*	2429	6.6	Q1
4	*Clinical Psychology Review*	2075	12.8	Q1
5	*Archives of Suicide Research*	1924	2.8	Q3
6	*Journal of Consulting and Clinical Psychology*	1885	5.9	Q1
7	*Psychological Medicine*	1796	6.9	Q1
8	*Journal of The American Academy of* *Child and Adolescent Psychiatry*	1674	13.3	Q1
9	*Journal of Abnormal Psychology*	1527	4.6	Q2
10	*American Journal of Psychiatry*	1405	17.7	Q1

### Publication analysis based on the authors

**Figure 5:**
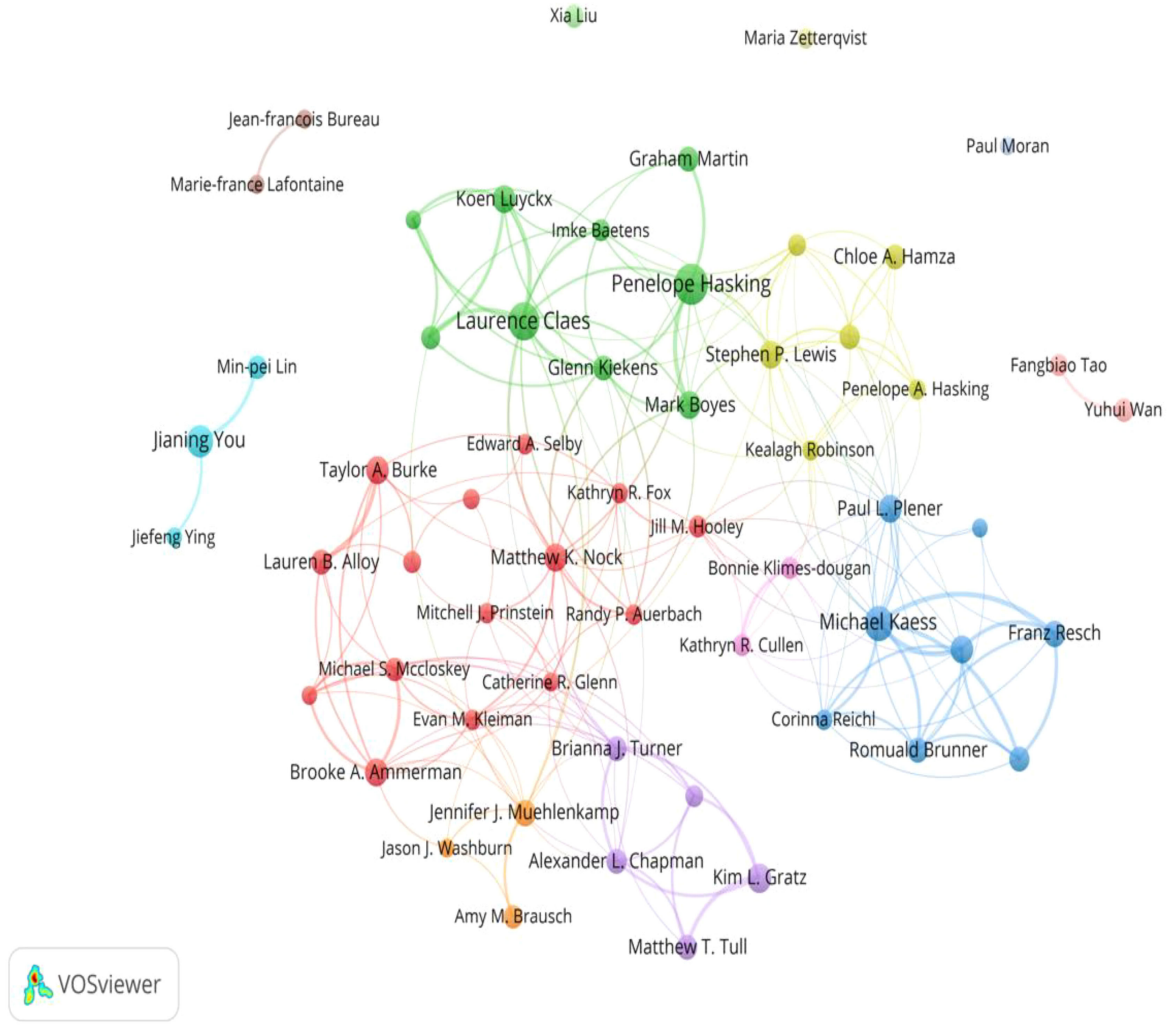
The co-authorship network of authors.

A total of 7239 authors published articles related to NSSI in adolescents. The visualization presented in **Figure 5** depicts the authorship network constructed using the VOSviewer software. The largest node is Penelope Hasking, and within the same cluster, strong connections can be observed, such as the partnership between Penelope Hasking and Mark Boyes. According to **Figure 5**, clusters lack interconnections with others, suggesting potential opportunities for further collaborative endeavors among these authors in the field of adolescent NSSI. **Table 5** showcases the top 10 productive authors. Penelope Hasking is the most prolific contributor, with 68 publications, followed by Laurence Claes (n = 58) and Michael Kaess (n = 45). Co-citation analysis refers to the phenomenon wherein a third author simultaneously cites the scholarly works of two authors. **Table 6** showcases the top 10 cited authors. Based on these findings, Matthew K. Nock stands out as the author with the highest frequency of co-citations, followed by E. David Klonsky and Kim L. Gratz.

**Table 5:** The top 10 productive authors.

Ranking	Author	Publications
1	Penelope Hasking	68
2	Laurence Claes	58
3	Michael Kaess	45
4	Jianing You	39
5	Kim L. Gratz	31
6	Mark Boyes	28
7	Julian Koenig	28
8	Paul L. Plener	28
9	Brooke A. Ammerman	26
10	Taylor A. Burke	26

**Table 6:** The top 10 cited authors.

Ranking	Cited Author	Citations
1	Matthew K. Nock	2847
2	E. David Klonsky	2094
3	Kim L. Gratz	1100
4	Janis Whitlock	884
5	Jennifer J. Muehlenkamp	789
6	Keith Hawton	615
7	Catherine R. Glenn	570
8	Laurence Claes	557
9	Paul L. Plener	557
10	Sarah V. Swannell	535

### Publication analysis based on keywords

**Figure 6:**
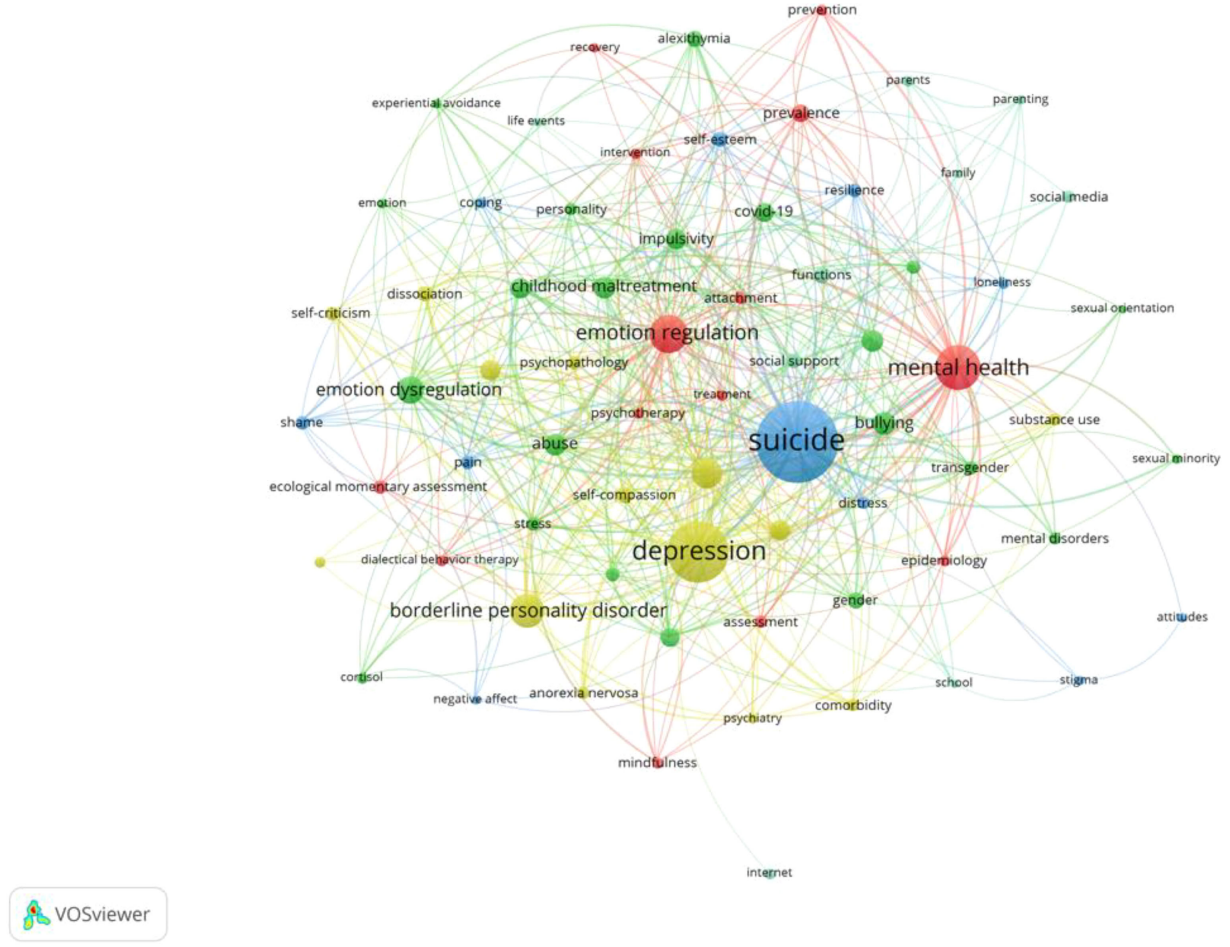
The network map of high-frequency keywords.

**Figure 7:**
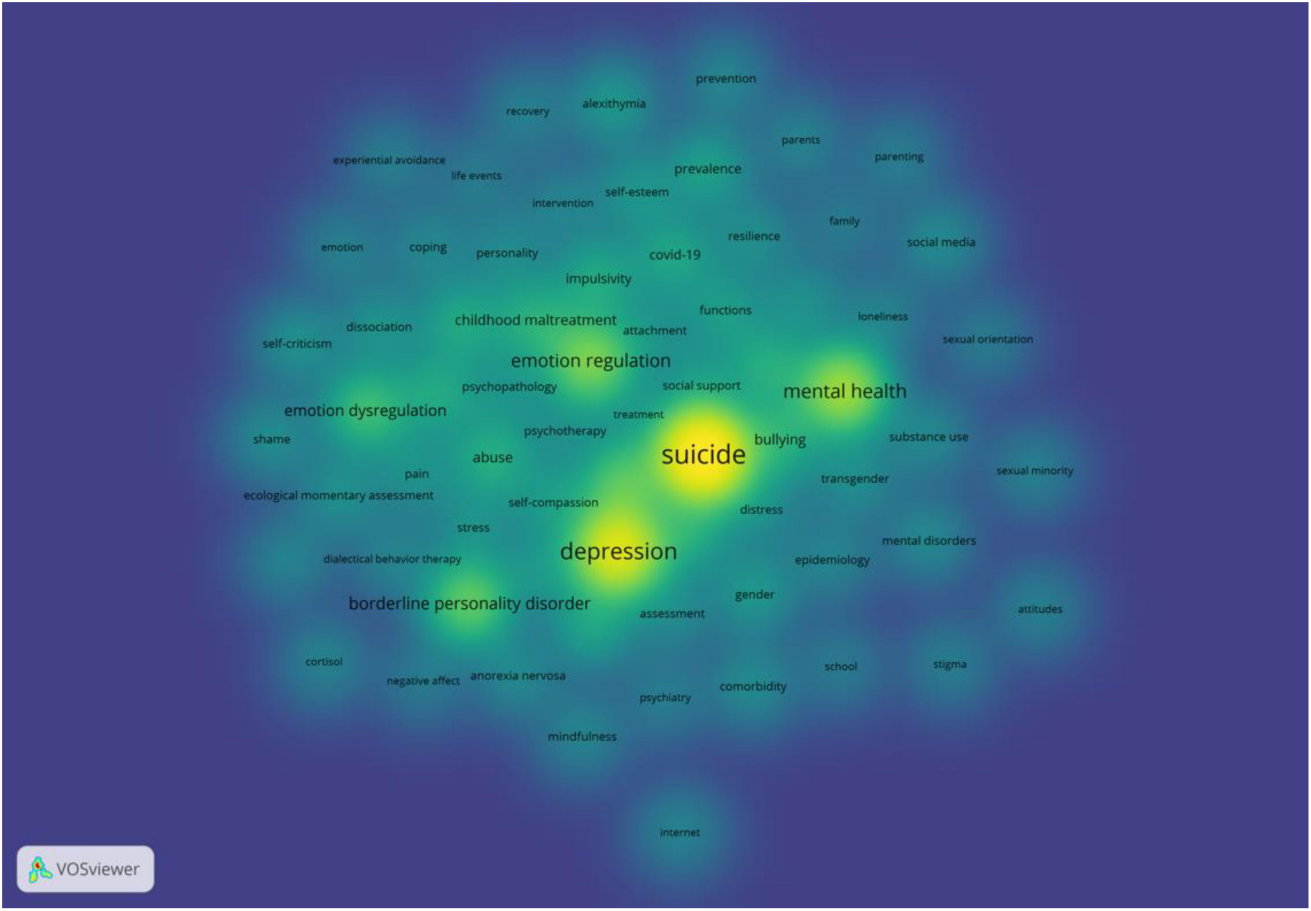
The density map of high-frequency keywords.

**Figure 8:**
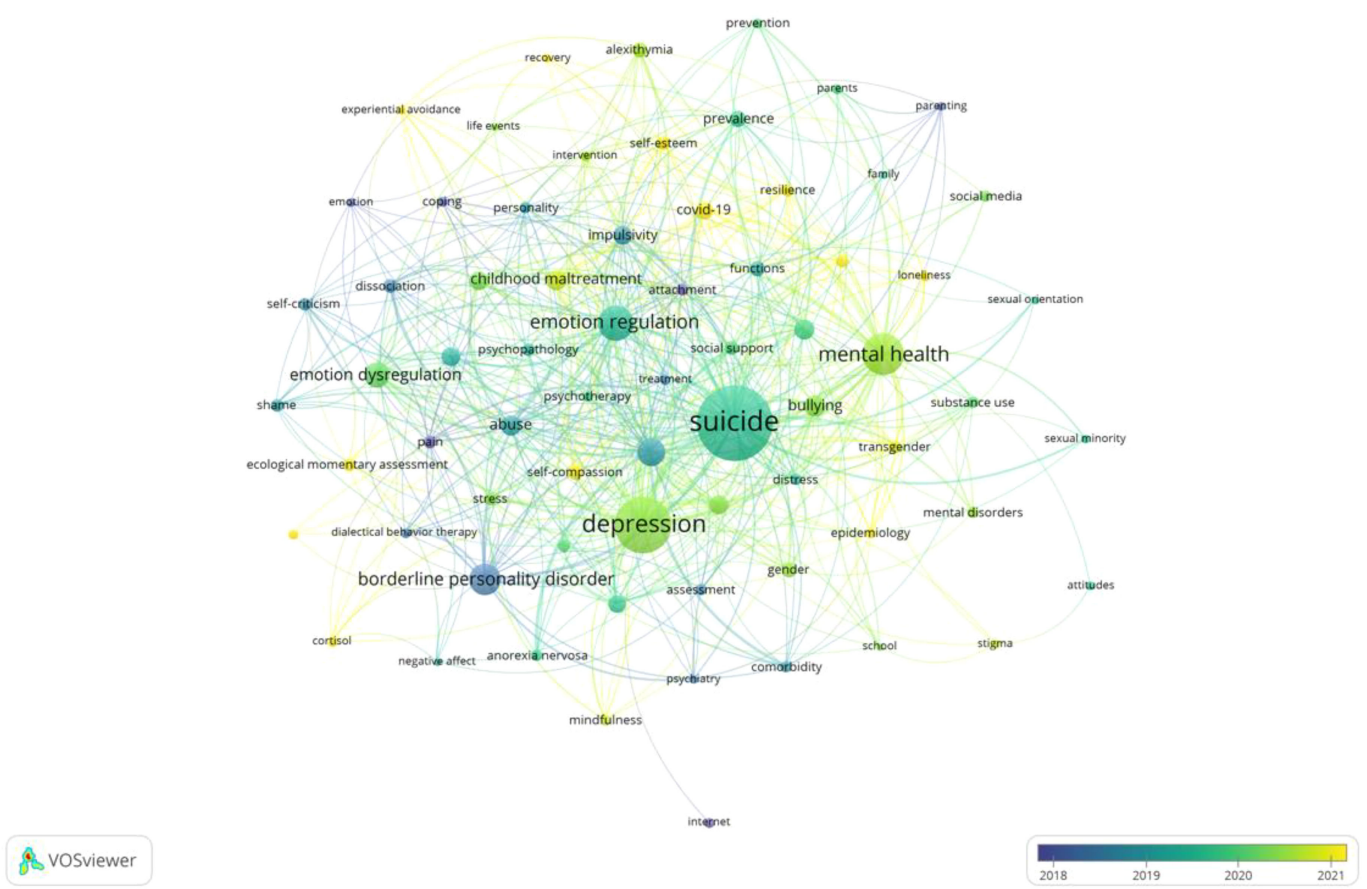
The overlay visualization of high-frequency keywords.

An analysis of the studies on NSSI in adolescents yielded 3211 keywords. The VOSviewer software was employed to generate a network visualization of co-occurring keywords and set the minimum number of occurrences of the keywords to 10 after merging synonyms and removing meaningless words to get 70 keywords. (**Figure 6**). The keywords were divided into five clusters, each represented by a distinct color in the diagram. The keywords in each cluster are listed in **Table 7**. Additionally, **Figure 7** shows the density map of keywords, in which the depth of color corresponds to the frequency of occurrence, highlighting the 8 keywords that appeared at least 50 times. **Table 8** lists the specific frequency of highlighted keywords in **Figure 7**. The most frequently occurring keyword was “suicide” (n = 293), followed by “depression” (n = 187), “mental health” (n = 119), “emotion regulation” (n = 92), and “ borderline personality disorder” (n = 76). Furthermore, **Figure 8** shows the result of the overlay visualization of keywords.

**Table 7:** Cluster analysis of keywords.

Cluster ID	Main Keywords (Occurrences)
Cluster 1 (red)	mental health (119), emotion regulation (92), prevalence (29), ecological momentary assessment (19),et al.
Cluster 2 (green)	emotion dysregulation (56), bullying (41), childhood maltreatment (40), abuse (39), et al.
Cluster 3 (blue)	suicide (293), self-esteem (22), pain (20), resilience (18), et al.
Cluster 4 (yellow)	depression (187), borderline personality disorder (76), eating disorder (65), anxiety (36), et al.
Cluster 5 (purple)	functions (25), social support (22), social media (17), parents (13), et al.

**Table 8:** The top 8 keywords of NSSI in adolescents.

Ranking	Keywords	Frequency
1	suicide	293
2	depression	187
3	mental health	119
4	emotion regulation	92
5	borderline personality disorder	76
6	eating disorder	65
7	emotion dysregulation	56
8	bullying	41

These keywords representing the research hotspots and emerging directions in the field of NSSI on adolescents. In **Figure 6**, Cluster 2 (green) shows researches on the etiology and risk factors of NSSI in adolescents, “bullying,” “emotion dysregulation,” and “childhood maltreatment,” all of them are important risk factors. Cluster 3 (blue) highlights the repercussions of NSSI on adolescents, with “suicide” emerging as the most prevalent consequence among them (3); Cluster 4 (yellow) illustrates the correlation between NSSI and other psychiatric comorbidities, encompassing “depression,” “borderline personality disorder,” and “eating disorder” as frequent comorbidities associated with NSSI (23); Cluster 1 (red) displays the intervention measures for NSSI in adolescents, “emotion regulation” and “ecological momentary assessment” as common interventions, while “mental health” and “prevalence” served as evaluation criteria; Cluster 5 (purple) demonstrates the trend of interdisciplinary research, where “social media” and “social support” are important topics in sociology and psychology. The keywords in Cluster 1 and Cluster 5 are shown in **Figure 8** as yellow-green and yellow colors, indicating that their corresponding average years of occurrence are relatively late. They will be a new direction in this field of NSSI in adolescents.

### Publication analysis based on burst detection

**Figure 9:**
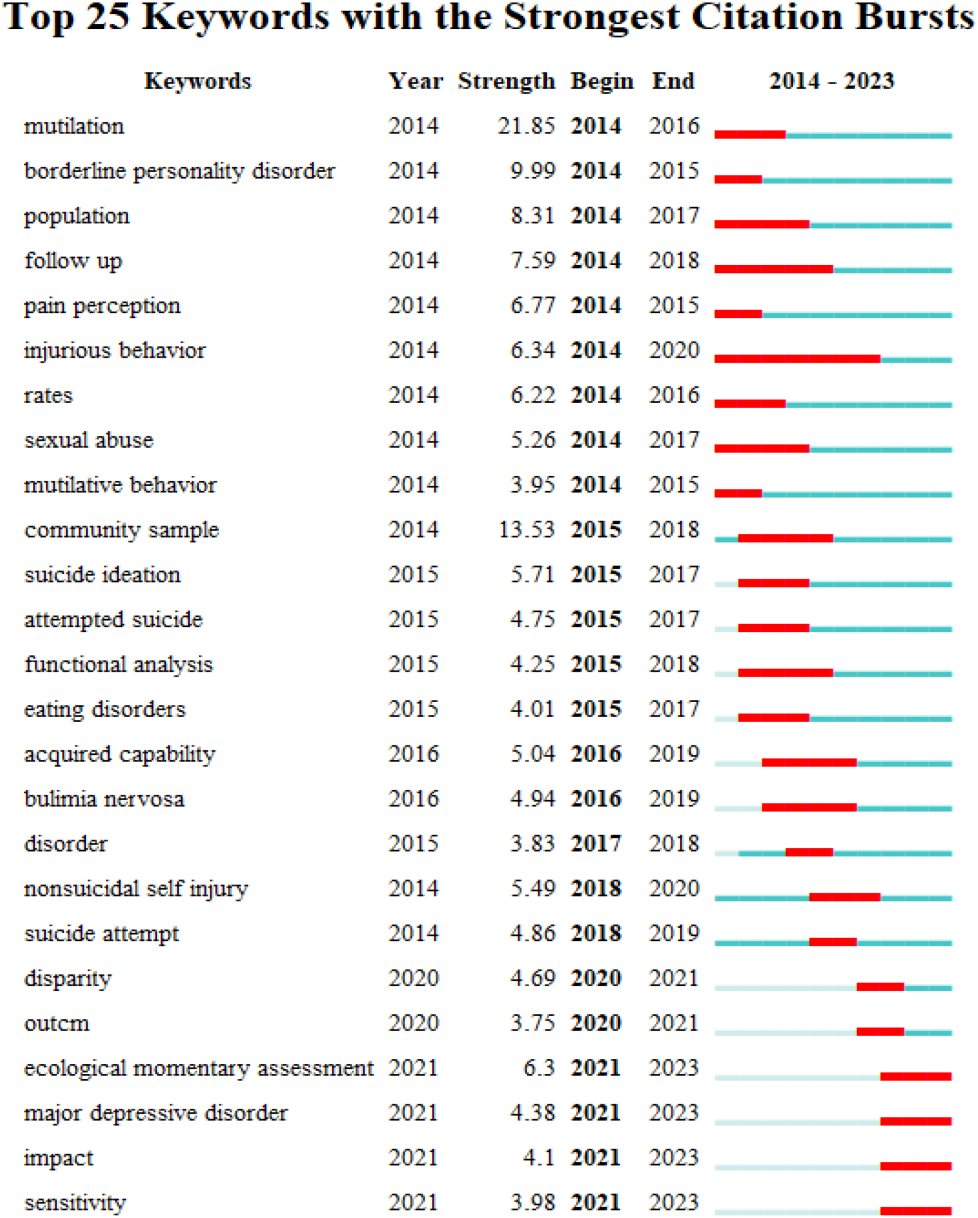
Top 25 Keywords with the Strongest Citation Bursts.

Keyword burst detection was carried out on 2177 articles to predict future trends in research. **Figure 9** shows the top 25 keywords with the strongest burst, listed in order of ‘outbreak’ year. The timeline is depicted as a blue line, where the time of the outbreak is shown as a red segment, indicating the duration of the outbreak. For each keyword, the figure showed the period the burst occurred and its corresponding strength. Concerning timing, during the analysis period (2014–2023), the first burst was identified as the keyword “mutilation”. In contrast, the keyword “injurious behavior” was shown to have the longest longevity, with its burst lasting for 7 years. With regard to burst intensity, **Figure 9** shows that for the period under analysis, the five keywords with the highest burst strength were ‘mutilation,’ “community sample,” “borderline personality,” “population,” and “follow up” (with values of strength 21.85, 13.53, 9.99, 8.31 and 7.59, respectively). “Ecological momentary assessment,” “major depressive disorder,” “impact,” and “sensitivity” have all come to prominence since 2023, and these words are currently the focus of discussion.

### Publication analysis based on co-cited references

A total of 54567 references were co-cited. **Table 9** presents the top 10 co-cited references pertaining to NSSI among adolescents, each garnering over 370 co-citations. The most frequently co-cited reference, published by Sarah V. Swannell et al. in 2014, investigated the methodological factors contributing to heterogeneity in prevalence estimates of NSSI, examining temporal effects and overall international prevalence. The second most co-cited publication, by Matthew K. Nock et al. in 2010, presents a comprehensive theoretical framework elucidating the development and maintenance of NSSI, synthesizing previous empirical findings, and proposing testable hypotheses for future research endeavors. The third highly co-cited reference, published by E. David Klonsky et al. in 2007, offers a comprehensive examination of the functions of deliberate self-injury by collating empirical studies, including self-reported motivations, phenomenological descriptions, and laboratory investigations exploring the effects of NSSI proxies on emotional and physiological arousal. Notably, these seminal references play a pivotal role in shaping the knowledge flow network within the field of NSSI in adolescents research.

**Table 9:** The top 10 co-cited references.

Ranking	Co-cited reference	Frequency
1	Swannell SV, 2014, suicide life-threat, v44, p273, doi 10.1111/sltb.12070	535
2	Nock MK, 2010, annu rev clin psycho, v6, p339, doi 10.1146/annurev.clinpsy.121208.131258	427
3	Klonsky ED, 2007, clin psychol rev, v27, p226, doi 10.1016/j.cpr.2006.08.002	413
4	Nock MK, 2006, psychiat res, v144, p65, doi 10.1016/j.psychres.2006.05.010	394
5	Nock MK, 2004, j consult clin psych, v72, p885, doi 10.1037/0022-006x.72.5.885	385
6	Muehlenkamp Jennifer J., 2012, child adolesc psychiatry ment health, v6, p10, doi 10.1186/1753-2000-6-10	346
7	Nock MK, 2009, curr dir psychol sci, v18, p78, doi 10.1111/j.1467-8721.2009.01613.x	331
8	Chapman AL, 2006, behav res ther, v44, p371, doi 10.1016/j.brat.2005.03.005	300
9	Klonsky ED, 2009, j psychopathol behav, v31, p215, doi 10.1007/s10862-008-9107-z	293
10	Gratz KL, 2001, j psychopathol behav, v23, p253, doi 10.1023/a:1012779403943	283

## Discussion

### General information

This study used visual tools to analyze 2177 articles in the field of NSSI in adolescents. Over the past decade, the growth trajectory has fluctuated over time, rapid growth since 2018. It is foreseeable that the accumulation of literature in this field will continue to increase in the future. The number of citations of articles published in this field has also increased exponentially, among which the top 10 literatures were cited more than 370 times each, which deserves the attention of more editors and researcher. The USA and its institutions, Harvard University, are the most influential countries and institutions. The number of NSSI in adolescents researches published by developed countries were significantly higher than that of developing countries, and the cooperation between developed countries was closer than that of developing countries. This might be related to the advantages of developed countries in technology, education, and economic fields. In the future, developing countries should strengthen learning, communication and cooperation with developed countries. The top 10 journals have published 604 articles, accounting for 27.7% of the total. Out of these top 10 journals, 7 are related to psychiatry, reflecting the insufficient interdisciplinary research in this field. Penelope Hasking was the most prolific contributor and Matthew K. Nock was the most influential author. They are the backbone of the field, and future researchers can build on these authors to take their research to new heights.

### Research topics and emerging trends

Keywords can reflect the core ideas of an article, and commonly used keywords are typically identified during the analysis process to pinpoint research hotspots within a field (24). Through the frequency analysis and clustering analysis of keywords, we identified some noteworthy aspects:

The association between NSSI and other psychiatric comorbidities was a hot area in the field of adolescent NSSI. Depression, borderline personality disorders, anxiety disorders, stress-related disorders, and eating disorders were the most common comorbidity topics. Adolescents who have comorbidities typically exhibit more severe mental symptoms and are likely to face a poorer prognosis (3). The potential interplay of mechanisms among comorbidities may be the cause of poor prognosis. A study pointed out that interpersonal relationships, emotional abuse, and depression degree of adolescent depression patients were closely related to the occurrence of NSSI (25). Another study from South Korea found that the combination of alcohol use disorder, rumination symptoms, depressive disorder, history of suicide attempts, and purging behavior were impact for NSSI in adolescent patients with eating disorders. There were also studies focusing on treatment measures for comorbidities (26). A randomized clinical trial (RCT) study found that Dialectical Behavior Therapy (DBT) interventions conducted by therapists trained in DBT suicide risk assessment and management plan were effective in reducing suicide attempts and NSSI attacks in adolescent patients with borderline personality disorder (27). Despite the numerous studies conducted on the comorbidity mechanisms and intervention measures for NSSI in adolescents, there are still notable gaps in knowledge when compared to the high incidence rate of severe comorbidities associated with NSSI. Researchers can continue to pay attention to this field in the future.

Etiology and risk factors were another hot topic in the field of adolescent NSSI. Researchers have always aimed to explore the etiology of NSSI from a neurobiological perspective. A study from Germany found that NSSI acts could be associated with a momentary increase of β-endorphin, which might reinforce NSSI engagement (28). A review identified that non-suicidal behaviors are related to top-down and bottom-up neural alterations (29). In addition, scholars have continued to identify risk factors for NSSI. This research field encompasses many areas, with the exploration of risk and protective factors such as NSSI physiology and psychology, as well as growth environment, family, personality traits, gender, etc., being the most prominent directions. Childhood maltreatment (30), bullying (31), emotion dysregulation (32), alexithymia (33), aggression (34), and other behaviors are risk factors for adolescent NSSI. Recently, the influence of sexual minorities or transgender groups (35), the spread of NSSI behavior through social media platforms such as YouTube and Twitter (36), or among peers and friends (37) have become increasingly popular in this field. Exploring the etiology and risk factors of NSSI in adolescents is current and is expected to remain the hottest research area for adolescents NSSI for a long time.

The impact of NSSI on adolescents was also a hot topic in this field. The psychological functions, impacts on patients, and roles in suicide and suicidal ideation of NSSI are the most common themes in this field. Intrapersonal functions, especially those concerning emotion regulation, represent the most prevalent psychological function associated with NSSI (38). The impacts of NSSI behaviors on patient life also include many aspects. Half of those who engage in self-injury report permanent scarring (39), and these individuals have negative perceptions of scars and feelings of shame (40). An initial psychometric validation study also found that those with negative perceptions of self-injury scars showed higher levels of social anxiety, depression, and even suicidal thoughts (41). Notably, NSSI can also adversely affect family and interpersonal relationships (42). NSSI also represents a risk factor for later suicidal behaviors (33). In the future, the mechanisms related to the impacts of NSSI on adolescents can be further explored.

According to visual analysis, intervention measures for NSSI in adolescents will be one of the hot topics in future research. A systematic review indicated that six specific psychotherapeutic interventions decreased NSSI in adolescents: Intensive Contextual Treatment (ICT), Therapeutic Assessment (TA), Emotional Regulation Individual Therapy for Adolescents (ERITA), Developmental Group Psychotherapy (DGP), Treatment for Self Injurious Behaviors (T-SIB) and Cutting Down Program (CDP) (43). In addition to specific psychotherapeutic interventions, pharmaceutical interventions are a research direction of NSSI adolescent interventions. A RCT study in China indicated that sertraline treatment can reduce the frequency of NSSI behaviors in adolescents (44). Physical interventions are also a common intervention measure. A recent study found that yoga is associated with reduced NSSI behaviors (45). Nevertheless, whether it is specific psychotherapeutic, physical, or pharmaceutical interventions, well-controlled studies still lack investigating treatment efficacy for NSSI in adolescents. Future investigators can focus more on intervention for NSSI in adolescents and conduct more research to replicate and confirm the results.

In recent years, research on NSSI has no longer been limited to psychiatry. Other disciplines, such as psychology and sociology, are also closely related to NSSI patients, and an increasing number of interdisciplinary studies have emerged. The research on interdisciplinary terms such as “rumination, “ “personality traits,” and “social support” is gradually emerging. A systematic review and meta-analysis found that rumination was associated with NSSI; thus, techniques focusing on rumination may benefit individuals engaging in NSSI (46). A clinical study has also shown that lower levels of social support are associated with NSSI in adolescents (47). In the future, the speed of information transmission will accelerate, and interdisciplinary communication and cooperation will become increasingly common. This is especially true of research on NSSI in adolescents, where interdisciplinary research will be a hot topic in the field.

In addition, the transformation of research methods was captured as follows via bursts: “rate” (2014–2016) and “population” (2014–2017) showed that a large number of demographic incidence rates and other investigative articles appeared at this stage; “community sample” (2015–2018) and “functional analysis” (2015–2018) indicated that this stage of research focused on the community rather than being limited to patients, and began to explore the physiological and psychological effects of NSSI behaviors on patients; “ecological momentary assessment”(2021-2023) suggested that research assessment methods may be more immediate and situational, allowing for more accurate capture of the state and trigger factors of NSSI in adolescents. Ecological Momentary Assessment (EMA) involves the repeated sampling of participants’ experiences and behaviors in real-time (48). Nowadays, mobile phones and web applications are the most commonly used tools in EMA, making EMA a suitable research tool for the population of NSSI in adolescents (49). Simultaneously, self-monitoring behaviors can reduce the frequency of their NSSI behaviors occurrence (50). As such, a study tried to find the risk factors of NSSI that could be targeted in interventions using EMA (51). Thus, EMA provides a promising new method for studying the NSSI in adolescents.

### Limitations

This study has some noted limitations and deficiencies. First, the data source is limited to the WoSCC database, which may not fully cover all studies relevant to NSSI in adolescents. Second, the literature selection criteria limited the language, and only English articles were selected for analysis, excluding research literature in other languages. Third, the time frame is limited to January 1, 2014, to December 31, 2023, which may limit understanding of the latest research trends. Fourthly, the literature selection criteria limited the types of articles and only selected relevant original articles and reviews for analysis, excluding other types of articles. Considering these limitations, future studies can use a wider range of data sources, including multiple languages, types of literature, longer time horizons, and multiple data analysis tools to obtain comprehensive and accurate results.

## Conclusions

In conclusion, the literature on NSSI in adolescents has significantly increased over the past decade. Through the visual analysis conducted by using CiteSpace and VOSviewer, we identified close collaborations among countries, institutions, and authors within this field. Moreover, the main current research trends in NSSI in adolescents were identified: the association between NSSI and other psychiatric comorbidities, the interaction between comorbidities, etiology and risk factors of NSSI in adolescents, and the impact of NSSI in adolescents. Simultaneously, intervention measures and interdisciplinary research on NSSI in adolescents will be hot topics in future research. We hope that this study can aid researchers in grasping the developmental characteristics of this field, providing valuable insights for their future endeavors. Ultimately, we aim to contribute to a deeper understanding and more effective support for adolescents struggling with NSSI.

## Data Availability

The original contributions presented in the study are included in the article/supplementary material. Further inquiries can be directed to the corresponding author.
